# The effects of family support and gender on mature student engagement in higher education

**DOI:** 10.3389/fpsyg.2015.00156

**Published:** 2015-02-17

**Authors:** Baljit Gill, Sarah Hayes, Carl Senior

**Affiliations:** ^1^Department of Psychology, Centre for Learning Innovation and Professional Practice, Aston UniversityBirmingham, UK; ^2^Department of Psychology, School of Life and Health Sciences, Aston UniversityBirmingham, UK

**Keywords:** mature, students, family, gender, university

While the factors driving the engagement of female mature students in the higher education sector (HE) have been extensively studied, e.g., Carney-Compton and Tan (2013) little work, if any, has been carried out examining the factors that drive engagement of their male counterparts. In light of the recent calls by various government-based think-tanks to expand the mature student population within HE, there is an obvious and urgent need to address this gap in our understanding (Bekhradnia, 2007; Sastry and Bekhradnia, 2007). Here the experiences of mature students at university, and the unique impact gender may have in influencing their initial motivation to enter HE, the responsibilities they bear, and the support they enjoy, are examined. While gender may underpin significantly diverse experiences of men and women at university, such diversity may have implications for the sector and the subsequent redesign of effective pedagogy.

Within the UK the Widening Participation agenda actively promotes the increased engagement of “non-traditional” learners in HE, and as such there has been a sharp increase in the participation of older students, with these mature learners embarking on higher level learning from a wide range of backgrounds and bringing with them a breadth of experiences (Thomas, 2001). However the rapid growth associated with the mature student population has brought with it a clarion call to ensure that HE provision is appropriately redesigned to ensure that these unique students engage fully with their learning experience at university. Such reflection is imperative as engagement as a mature student can have a transformative impact on the lives of not just the student but their families as well (Wainwright and Marandet, 2010). To this end, a significant amount of research has explored the impact of family on the mature student experience, however as alluded to above, such research focusses primarily on the experiences of mature female students, extensively and ostensibly documenting the barriers that women face in accessing and progressing through their studies (Edwards, 1993; Reay et al., 2002; McGivney, 2003). The pervasive emphasis of understanding the factors that drive the engagement of female students is perhaps quite unfortunate, especially in light of the fact that familial motivations are now seen as the primary facilitator for education engagements in male pupils across the diversity of racial and cultural boundaries (Kenny and Donaldson, 1991; Gloria et al., 2005). By and large such work has been primarily embedded within the secondary education sector and as these pupils transition into HE students, it is likely that the significance of such extrinsic motivations will remain. Yet surprisingly little is known of the effects of familial motivations on male mature student populations. While key sociological research in this area has identified the need for familial support when encountering a change in social identity that the mature student inevitably goes through as they progress through their respective programmes of study (Baxter and Britton, 2001), such work is predominantly focussed on the experiences of female mature students and as such research is needed to fully explicate the experience and the impact, if any, of family on the male student experience.

The motivation displayed by older learners to engage with a university programme of study has been the focus of a number of studies which have identified the vocational drivers for many mature students, as well as exploring the sense of unfulfilled potential often borne by those who opted to return to education (Britton and Baxter, 1999). While intrinsic motivation appears to be key in both the decision-making and subsequent success of older learners (Murphy and Roopchand, 2003; McCune et al., 2010), the decision to return to learning is often generated by key life transitions, whether situational events such as unemployment or divorce, or dispositional aspects of personal satisfaction; all key influencers on family wellbeing. Here, individuals enter HE as a mechanism to redress the balance in an unsatisfactory life, both individually and for their families (Dawson and Boulton, 2000). Motivation may be gendered; male students are labor-market focussed, with an aspiration to better provide materially for their family, while female students may seek personal improvement to offer inspirational role models for their children (Marks et al., 2003). Recent studies have further explored the power that education has as a tool for social mobility and inter-generational learning, with mature learners expressing a desire to promote higher aspirations in their own children by embarking on a programme of study themselves (Wainwright and Marandet, 2010).

It goes without saying that the transition to university is not a solitary experience for the mature student and is one that does impact the whole family unit (Kantanis, 2002). Here the various reactions of family, which can range from encouragement through to mockery, envy and resentfulness, impact significantly on the transition of the individuals into their new role as a mature student. This study found that the level of support offered was mediated by the value placed on education by the immediate family and friends as well as the understanding of the student's motivation for returning to education. Familial support was further influenced by the level of disruption that the family would incur; indeed, unwillingness in general to cause change was identified in a study exploring the reasons for the non-participation of potential older learners. where the individual's reliance on their informal networks of family and friends instilled a reluctance to disrupt the status quo (Fuller and Heath, 2010).

The familial influence on mature students is inextricably linked to gendered roles within the family, notably the traditionally female role of carer in contrast to the conventional male role of provider. Studies have highlighted the barriers and challenges faced by women with child caring responsibilities (Leppel, 2002). Their situation has tended to be problematized, identifying the conflict between the role of carer and the role of student, with the two identities at odds with each other (McGivney, 1999). Arguably men are better able to separate their personal and domestic lives from their academic and career aspirations with significantly more support from their partners (Tett, 2000). The vital importance of support has been stressed in an extensive study exploring the “public” and the “private” lives of female mature students, which found that the greatest emotional toll was felt by women with children and an unsupportive family, often compounded by cultural expectations (Edwards, 1993). The clear correlation between levels of support, satisfaction, stress and self-esteem suggests that male students may benefit significantly from the higher level of support they receive, thus impacting their engagement with HE (Norton et al., 1998; Winn, 2002). As is evident, much of this research on support that men may benefit from is a little dated; this warrants renewed investigation, to assess the experiences of male students today.

The decision to return to learning as a mature student is significantly mediated by other factors such as cultural and socio-economic background, the impact of which is inextricably linked to gender (Baxter and Britton, 2001). Becoming a student not only challenges traditional gender roles and identity within the family, to be in education potentially challenges traditional male working class identity, threatening both an individual's sense of self and demanding renegotiation of their relationships with others. Take the finding that perceived social class plays a major role in the decision to become a mature student, sociological research shows that study in HE is not viewed as a working class activity, but is seen as self-indulgent and firmly entrenched in the traditional and masculine gender roles of the provider (Marks et al., 2003). This is inextricably linked with the connotations of being be a “good” parent. While women are deemed nurturers, offering emotional support for their offspring, men's roles are embedded in that of providing for their family in financial and material terms. Concepts of masculinity and its relationship with class and ethnicity can inform working men's decisions not to participate in a programme of study in HE, with the conceptualization of university students in a framework of negative, middle class masculinity significantly alien to their own lives (Archer et al., 2001).

For those adults considering returning to education as mature students, the decision-making process is often based on a costs and benefits analysis for the family unit and that may be mediated by gender (Davies et al., 2002). Potential mature students view the option of higher education as an immediate investment of time and money by not just the individual, but the family. Any potential benefits are seen as delayed and uncertain; future rewards versus debt or unemployment; personal achievement versus failure; time versus family and relationship obligations, and financial implications of supporting a family whilst studying (Davies and Williams, 2001). This raises the pertinent question of the worth of a degree, whether real or perceived; despite a report by the UK based University think-thank, the Million+ and LSE (2013) the rate of return on the investment of time and money can be variable, and may be influenced by age and gender. Non-economic benefits are, however, significant, in the form of intergenerational benefits and increased potential for social mobility of the family, although mature students may find it more difficult to gain graduate employment, with a lower rate of earnings growth and higher levels of job dissatisfaction, and the consequent impact on their families (Purcell et al., 2007).

This historical, and perhaps wholly understandable focus on the experiences of mature women learners, while illuminating the distinct experiences of this group, nevertheless leaves a gap in our understanding of the specific nature of mature men's experiences of higher education, with the danger of making assumptions about the changing nature of their participation based on historical research on women's experiences. Such studies that do exist incorporating the male experience tend to be rather dated, presenting us with only a limited understanding of their world. This has wide-reaching implications across the sector in how we support such students through their studies. There is a clear need to recognize the multiple identities displayed by male mature students; what it is in their decision-making, their social identity, and their role within the family, that hinders or underpins their choices, and how institutions can harness the positive aspects, diminish the influence of the impediments, and develop innovative support mechanisms. Levels of female participation have escalated across the HE sector in accordance with the specific support mechanisms that facilitate the engagement of female mature students. The time is ripe to consider whether these existing mechanisms are appropriate for men.

## Conflict of interest statement

The authors declare that the research was conducted in the absence of any commercial or financial relationships that could be construed as a potential conflict of interest.
